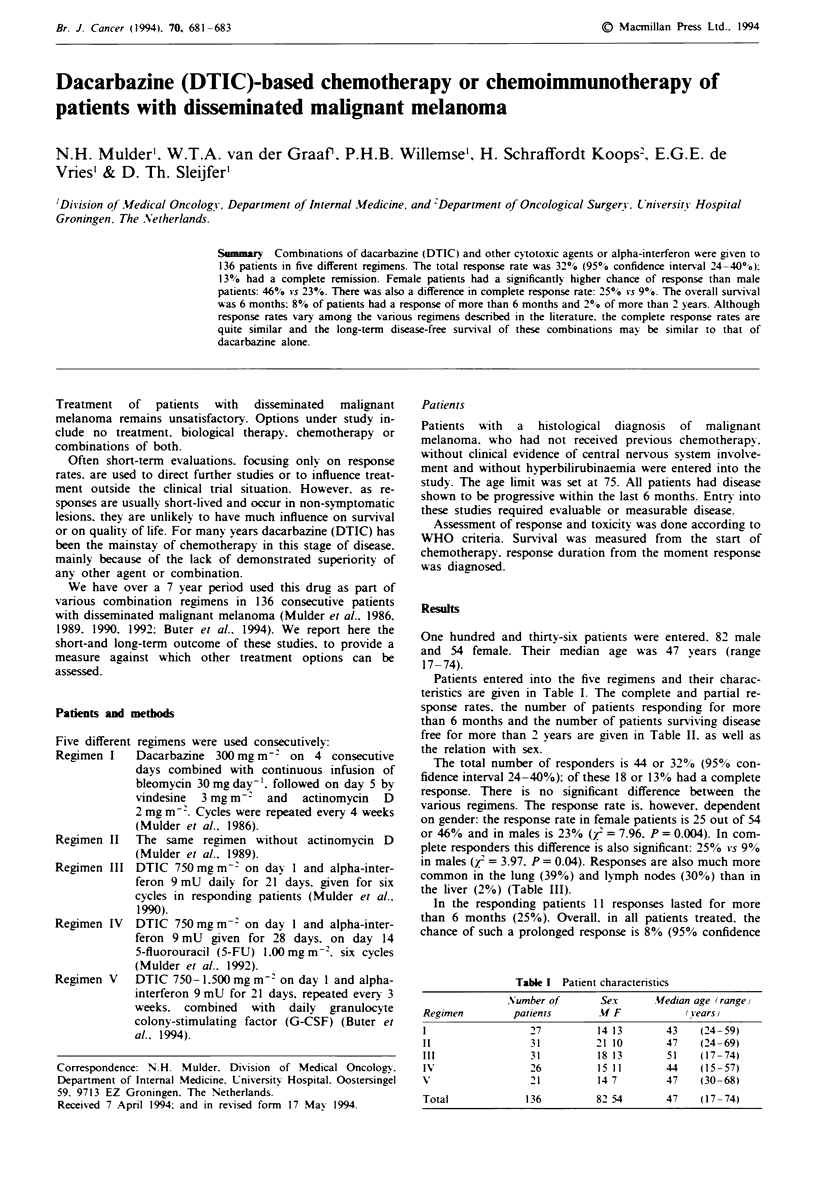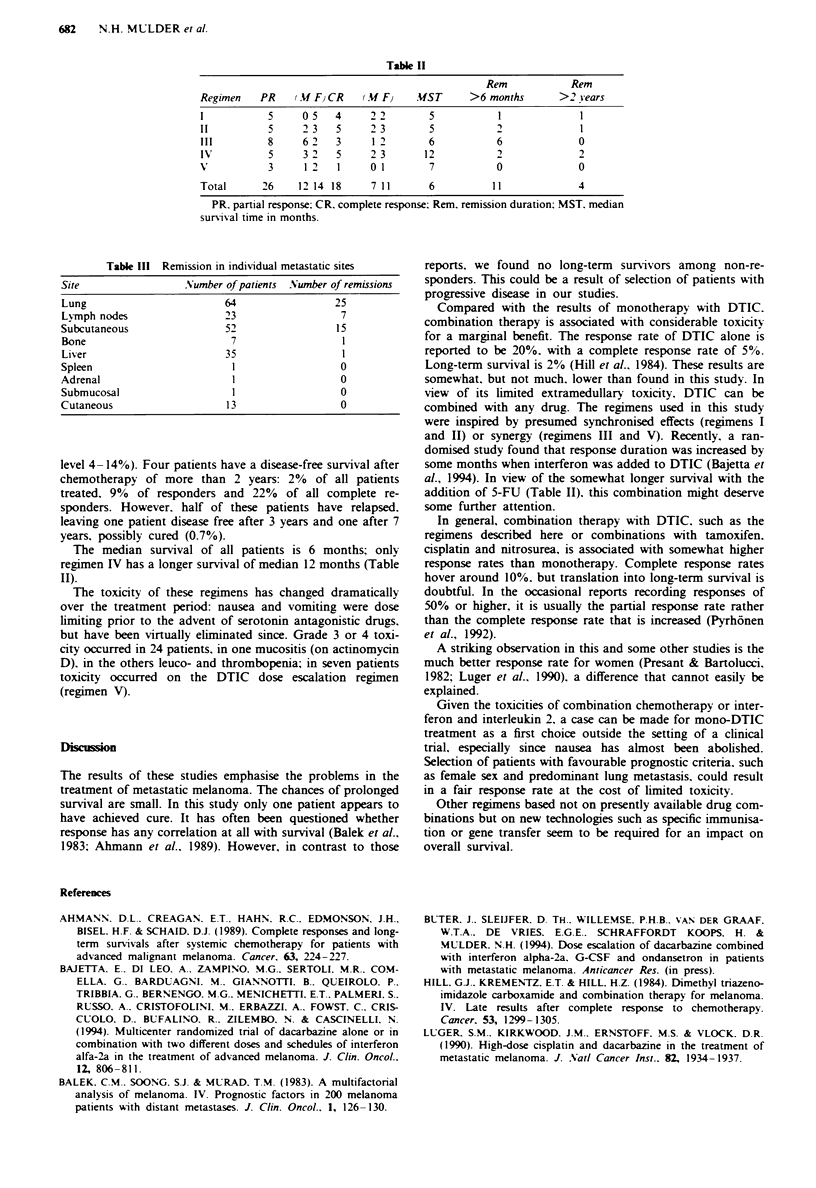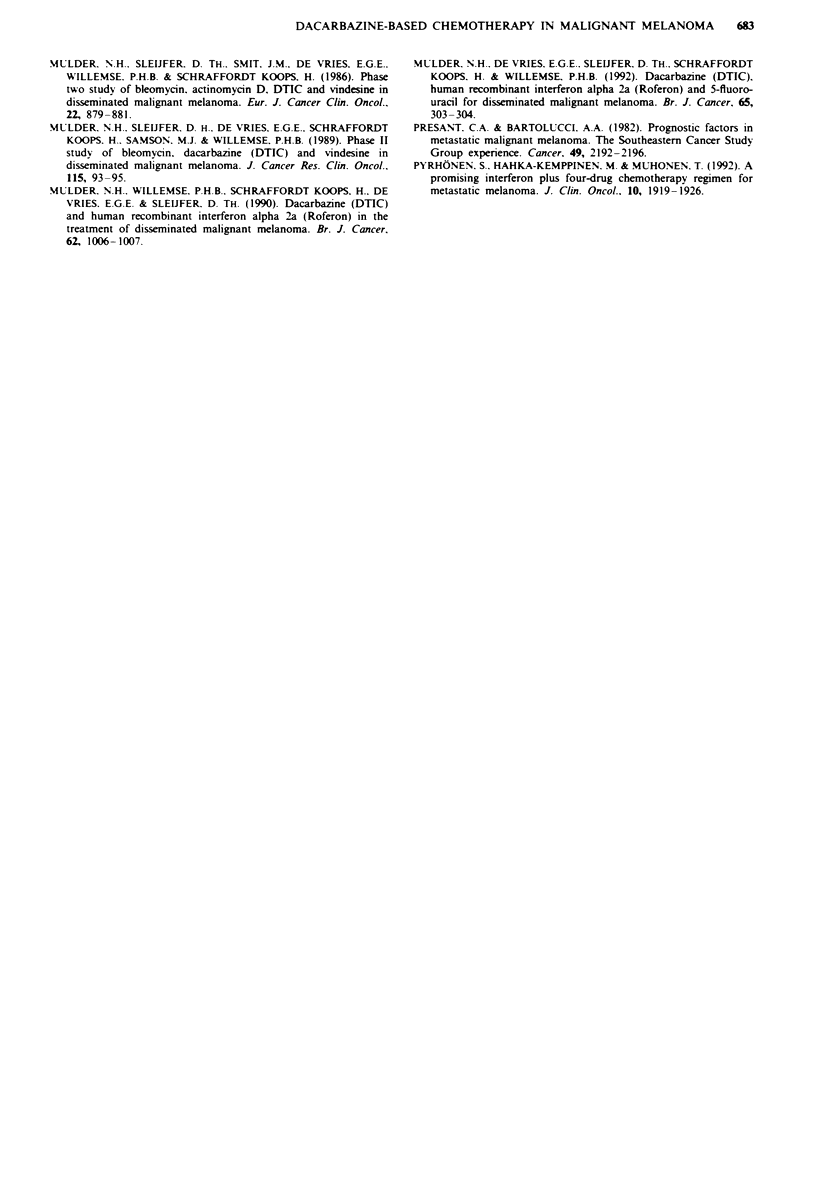# Dacarbazine (DTIC)-based chemotherapy or chemoimmunotherapy of patients with disseminated malignant melanoma.

**DOI:** 10.1038/bjc.1994.372

**Published:** 1994-10

**Authors:** N. H. Mulder, W. T. van der Graaf, P. H. Willemse, H. S. Koops, E. G. de Vries, D. T. Sleijfer

**Affiliations:** Department of Internal Medicine, University Hospital Groningen, The Netherlands.

## Abstract

Combinations of dacarbazine (DTIC) and other cytotoxic agents or alpha-interferon were given to 136 patients in five different regimens. The total response rate was 32% (95% confidence interval 24-40%); 13% had a complete remission. Female patients had a significantly higher chance of response than male patients: 46% vs 23%. There was also a difference in complete response rate: 25% vs 9%. The overall survival was 6 months; 8% of patients had a response of more than 6 months and 2% of more than 2 years. Although response rates vary among the various regimens described in the literature, the complete response rates are quite similar and the long-term disease-free survival of these combinations may be similar to that of dacarbazine alone.


### Dacarbazine (DTIC)-based chemotherapy or chemoimmunotherapy of patients with disseminated malignant melanoma

N.H. Mulder[1], W.T.A. van der Graaf[1], P.H.B. Willemse[1], H. Schraffordt Koops[2], E.G.E. de Vries[1] & D. Th. Sleijfer[1]

[1]*Division of Medical Oncology, Department of Internal Medicine, and* [2]*Department of Oncological Surgery, University Hospital Groningen, The Netherlands.*

**Summary** Combinations of dacarbazine (DTIC) and other cytotoxic agents or alpha-interferon were given to 136 patients in five different regimens. The total response rate was 32% (95% confidence interval 24-40%); 13% had a complete remission. Female patients had a significantly higher chance of response than male patients: 46% *vs* 23%. There was also a difference in complete response rate: 25% *vs* 9%. The overall survival was 6 months; 8% of patients had a response of more than 6 months and 2% of more than 2 years. Although response rates vary among the various regimens described in the literature, the complete response rates are quite similar and the long-term disease-free survival of these combinations may be similar to that of dacarbazine alone.

Treatment of patients with disseminated malignant melanoma remains unsatisfactory. Options under study include no treatment, biological therapy, chemotherapy or combinations of both.

Often short-term evaluations, focusing only on response rates, are used to direct further studies or to influence treatment outside the clinical trial situation. However, as responses are usually short-lived and occur in non-symptomatic lesions, they are unlikely to have much influence on survival or on quality of life. For many years dacarbazine (DTIC) has been the mainstay of chemotherapy in this stage of disease, mainly because of the lack of demonstrated superiority of any other agent or combination.

We have over a 7 year period used this drug as part of various combination regimens in 136 consecutive patients with disseminated malignant melanoma (Mulder *et al.*, 1986, 1989, 1990, 1992; Buter *et al.*, 1994). We report here the short-and long-term outcome of these studies, to provide a measure against which other treatment options can be assessed.

### Patients and methods

Five different regimens were used consecutively:

- Regimen I: Dacarbazine 300 mg $m^{-2}$ on 4 consecutive days combined with continuous infusion of bleomycin 30 mg $day^{-1}$, followed on day 5 by vindesine 3 mg $m^{-2}$ and actinomycin D 2 mg $m^{-2}$. Cycles were repeated every 4 weeks (Mulder *et al.*, 1986).
- Regimen II: The same regimen without actinomycin D (Mulder *et al.*, 1989).
- Regimen III: DTIC 750 mg $m^{-2}$ on day 1 and alpha-interferon 9 mU daily for 21 days, given for six cycles in responding patients (Mulder *et al.*, 1990).
- Regimen IV: DTIC 750 mg $m^{-2}$ on day 1 and alpha-interferon 9 mU given for 28 days, on day 14 5-fluorouracil (5-FU) 1.00 mg $m^{-2}$, six cycles (Mulder *et al.*, 1992).
- Regimen V: DTIC 750-1,500 mg $m^{-2}$ on day 1 and alpha-interferon 9 mU for 21 days, repeated every 3 weeks, combined with daily granulocyte colony-stimulating factor (G-CSF) (Buter *et al.*, 1994).

Correspondence: N.H. Mulder, Division of Medical Oncology, Department of Internal Medicine, University Hospital, Oostersingel 59, 9713 EZ Groningen, The Netherlands.
Received 7 April 1994; and in revised form 17 May 1994.

### *Patients*

Patients with a histological diagnosis of malignant melanoma, who had not received previous chemotherapy, without clinical evidence of central nervous system involvement and without hyperbilirubinaemia were entered into the study. The age limit was set at 75. All patients had disease shown to be progressive within the last 6 months. Entry into these studies required evaluable or measurable disease.

Assessment of response and toxicity was done according to WHO criteria. Survival was measured from the start of chemotherapy, response duration from the moment response was diagnosed.

### Results

One hundred and thirty-six patients were entered, 82 male and 54 female. Their median age was 47 years (range 17-74).

Patients entered into the five regimens and their characteristics are given in Table I. The complete and partial response rates, the number of patients responding for more than 6 months and the number of patients surviving disease free for more than 2 years are given in Table II, as well as the relation with sex.

The total number of responders is 44 or 32% (95% confidence interval 24-40%); of these 18 or 13% had a complete response. There is no significant difference between the various regimens. The response rate is, however, dependent on gender: the response rate in female patients is 25 out of 54 or 46% and in males is 23% ($\chi^2 = 7.96$, $P = 0.004$). In complete responders this difference is also significant: 25% *vs* 9% in males ($\chi^2 = 3.97$, $P = 0.04$). Responses are also much more common in the lung (39%) and lymph nodes (30%) than in the liver (2%) (Table III).

In the responding patients 11 responses lasted for more than 6 months (25%). Overall, in all patients treated, the chance of such a prolonged response is 8% (95% confidence

**Table I** Patient characteristics

| *Regimen* | *Number of patients* | *Sex M F* | *Median age (range) (years)* |
|---|---|---|---|
| I | 27 | 14 13 | 43 (24-59) |
| II | 31 | 21 10 | 47 (24-69) |
| III | 31 | 18 13 | 51 (17-74) |
| IV | 26 | 15 11 | 44 (15-57) |
| V | 21 | 14 7 | 47 (30-68) |
| Total | 136 | 82 54 | 47 (17-74) |

**Table II**

| Regimen | PR | (M F) | CR | (M F) | MST | Rem >6 months | Rem >2 years |
|---|---|---|---|---|---|---|---|
| I | 5 | 0 5 | 4 | 2 2 | 5 | 1 | 1 |
| II | 5 | 2 3 | 5 | 2 3 | 5 | 2 | 1 |
| III | 8 | 6 2 | 3 | 1 2 | 6 | 6 | 0 |
| IV | 5 | 3 2 | 5 | 2 3 | 12 | 2 | 2 |
| V | 3 | 1 2 | 1 | 0 1 | 7 | 0 | 0 |
| Total | 26 | 12 14 | 18 | 7 11 | 6 | 11 | 4 |

PR, partial response; CR, complete response; Rem, remission duration; MST, median survival time in months.

**Table III** Remission in individual metastatic sites

| Site | Number of patients | Number of remissions |
|---|---|---|
| Lung | 64 | 25 |
| Lymph nodes | 23 | 7 |
| Subcutaneous | 52 | 15 |
| Bone | 7 | 1 |
| Liver | 35 | 1 |
| Spleen | 1 | 0 |
| Adrenal | 1 | 0 |
| Submucosal | 1 | 0 |
| Cutaneous | 13 | 0 |

level 4–14%). Four patients have a disease-free survival after chemotherapy of more than 2 years: 2% of all patients treated, 9% of responders and 22% of all complete responders. However, half of these patients have relapsed, leaving one patient disease free after 3 years and one after 7 years, possibly cured (0.7%).

The median survival of all patients is 6 months; only regimen IV has a longer survival of median 12 months (Table II).

The toxicity of these regimens has changed dramatically over the treatment period: nausea and vomiting were dose limiting prior to the advent of serotonin antagonistic drugs, but have been virtually eliminated since. Grade 3 or 4 toxicity occurred in 24 patients, in one mucositis (on actinomycin D), in the others leuco- and thrombopenia; in seven patients toxicity occurred on the DTIC dose escalation regimen (regimen V).

### Discussion

The results of these studies emphasise the problems in the treatment of metastatic melanoma. The chances of prolonged survival are small. In this study only one patient appears to have achieved cure. It has often been questioned whether response has any correlation at all with survival (Balek *et al.*, 1983; Ahmann *et al.*, 1989). However, in contrast to those reports, we found no long-term survivors among non-responders. This could be a result of selection of patients with progressive disease in our studies.

Compared with the results of monotherapy with DTIC, combination therapy is associated with considerable toxicity for a marginal benefit. The response rate of DTIC alone is reported to be 20%, with a complete response rate of 5%. Long-term survival is 2% (Hill *et al.*, 1984). These results are somewhat, but not much, lower than found in this study. In view of its limited extramedullary toxicity, DTIC can be combined with any drug. The regimens used in this study were inspired by presumed synchronised effects (regimens I and II) or synergy (regimens III and V). Recently, a randomised study found that response duration was increased by some months when interferon was added to DTIC (Bajetta *et al.*, 1994). In view of the somewhat longer survival with the addition of 5-FU (Table II), this combination might deserve some further attention.

In general, combination therapy with DTIC, such as the regimens described here or combinations with tamoxifen, cisplatin and nitrosurea, is associated with somewhat higher response rates than monotherapy. Complete response rates hover around 10%, but translation into long-term survival is doubtful. In the occasional reports recording responses of 50% or higher, it is usually the partial response rate rather than the complete response rate that is increased (Pyrhönen *et al.*, 1992).

A striking observation in this and some other studies is the much better response rate for women (Presant & Bartolucci, 1982; Luger *et al.*, 1990), a difference that cannot easily be explained.

Given the toxicities of combination chemotherapy or interferon and interleukin 2, a case can be made for mono-DTIC treatment as a first choice outside the setting of a clinical trial, especially since nausea has almost been abolished. Selection of patients with favourable prognostic criteria, such as female sex and predominant lung metastasis, could result in a fair response rate at the cost of limited toxicity.

Other regimens based not on presently available drug combinations but on new technologies such as specific immunisation or gene transfer seem to be required for an impact on overall survival.

### References

AHMANN, D.L., CREAGAN, E.T., HAHN, R.C., EDMONSON, J.H., BISEL, H.F. & SCHAID, D.J. (1989). Complete responses and long-term survivals after systemic chemotherapy for patients with advanced malignant melanoma. *Cancer*, **63**, 224–227.

BAJETTA, E., DI LEO, A., ZAMPINO, M.G., SERTOLI, M.R., COMELLA, G., BARDUAGNI, M., GIANNOTTI, B., QUEIROLO, P., TRIBBIA, G., BERNENGO, M.G., MENICHETTI, E.T., PALMERI, S., RUSSO, A., CRISTOFOLINI, M., ERBAZZI, A., FOWST, C., CRISCUOLO, D., BUFALINO, R., ZILEMBO, N. & CASCINELLI, N. (1994). Multicenter randomized trial of dacarbazine alone or in combination with two different doses and schedules of interferon alfa-2a in the treatment of advanced melanoma. *J. Clin. Oncol.*, **12**, 806–811.

BALEK, C.M., SOONG, S.J. & MURAD, T.M. (1983). A multifactorial analysis of melanoma. IV. Prognostic factors in 200 melanoma patients with distant metastases. *J. Clin. Oncol.*, **1**, 126–130.

BUTER, J., SLEIJFER, D. TH., WILLEMSE, P.H.B., VAN DER GRAAF, W.T.A., DE VRIES, E.G.E., SCHRAFFORDT KOOPS, H. & MULDER, N.H. (1994). Dose escalation of dacarbazine combined with interferon alpha-2a, G-CSF and ondansetron in patients with metastatic melanoma. *Anticancer Res.* (in press).

HILL, G.J., KREMENTZ, E.T. & HILL, H.Z. (1984). Dimethyl triazenoimidazole carboxamide and combination therapy for melanoma. IV. Late results after complete response to chemotherapy. *Cancer*, **53**, 1299–1305.

LUGER, S.M., KIRKWOOD, J.M., ERNSTOFF, M.S. & VLOCK, D.R. (1990). High-dose cisplatin and dacarbazine in the treatment of metastatic melanoma. *J. Natl Cancer Inst.*, **82**, 1934–1937.

MULDER, N.H., SLEIJFER, D. TH., SMIT, J.M., DE VRIES, E.G.E., WILLEMSE, P.H.B. & SCHRAFFORDT KOOPS, H. (1986). Phase two study of bleomycin, actinomycin D, DTIC and vindesine in disseminated malignant melanoma. *Eur. J. Cancer Clin. Oncol.*, **22**, 879–881.

MULDER, N.H., SLEIJFER, D. H., DE VRIES, E.G.E., SCHRAFFORDT KOOPS, H., SAMSON, M.J. & WILLEMSE, P.H.B. (1989). Phase II study of bleomycin, dacarbazine (DTIC) and vindesine in disseminated malignant melanoma. *J. Cancer Res. Clin. Oncol.*, **115**, 93–95.

MULDER, N.H., WILLEMSE, P.H.B., SCHRAFFORDT KOOPS, H., DE VRIES, E.G.E. & SLEIJFER, D. TH. (1990). Dacarbazine (DTIC) and human recombinant interferon alpha 2a (Roferon) in the treatment of disseminated malignant melanoma. *Br. J. Cancer*, **62**, 1006–1007.

MULDER, N.H., DE VRIES, E.G.E., SLEIJFER, D. TH., SCHRAFFORDT KOOPS, H. & WILLEMSE, P.H.B. (1992). Dacarbazine (DTIC), human recombinant interferon alpha 2a (Roferon) and 5-fluorouracil for disseminated malignant melanoma. *Br. J. Cancer*, **65**, 303–304.

PRESANT, C.A. & BARTOLUCCI, A.A. (1982). Prognostic factors in metastatic malignant melanoma. The Southeastern Cancer Study Group experience. *Cancer*, **49**, 2192–2196.

PYRHÖNEN, S., HAHKA-KEMPPINEN, M. & MUHONEN, T. (1992). A promising interferon plus four-drug chemotherapy regimen for metastatic melanoma. *J. Clin. Oncol.*, **10**, 1919–1926.